# Oral melanoacanthoma: a case report and review of the literature

**DOI:** 10.1186/1752-1947-3-11

**Published:** 2009-01-13

**Authors:** Vidya Lakshminarayanan, Kannan Ranganathan

**Affiliations:** 1 Department of Oral and Maxillofacial Pathology, Ragas Dental College and Hospital, East Coast Road, Chennai, Tamilnadu 600119, India

## Abstract

**Introduction:**

Oral melanoacanthoma is a rare, benign pigmented lesion characterized clinically by the sudden appearance and rapid growth of a macular brown-black lesion and histologically by acanthosis of the superficial epithelium and proliferation of dendritic melanocytes.

**Case presentation:**

We present a case report of oral melanoacanthoma in a 24-year-old Asian Indian man. He presented with an intra-oral brown macular lesion on the left buccal mucosa with a duration of one and a half months. Microscopic examination revealed acanthosis of stratified squamous surface epithelium and dendritic melanocytes diffusely distributed in the epithelium; the Masson-Fontana silver impregnation technique was used to demonstrate the dendritic melanocytes. Based on the history, clinical features and histological presentation, the lesion was diagnosed as melanoacanthoma.

**Conclusion:**

This is the first reported instance of oral melanoacanthoma in the Indian sub-continent. This report details the course of the lesion from diagnosis to its resolution. Melanoacanthoma must be differentiated from other intra-oral pigmented lesions and biopsy may be required to rule out melanoma.

## Introduction

Melanoacanthoma of the oral mucosa is a rare condition indicative of a reactive process [1]. Oral melanoacanthoma was first reported in 1978 [2] and to the best of our knowledge, only 50 cases of melanoacanthoma have been reported in the literature to date (Table 1) [2-14]. The clinical presentation is a brown to brown-black macular lesion, predominantly solitary, encountered in the younger age group with a distinct female predilection [3,12]. The most common site affected is the buccal mucosa. Melanoacanthoma has been reported in labial mucosa, palate, gingiva, alveolar mucosa and oropharynx (Table 1). The typical histological picture of melanoacanthoma is the proliferation of dendritic melanocytes throughout the epithelium. The epithelium exhibits acanthosis and spongiosis. A chronic inflammatory cell infiltrate with eosinophils may be noted. The lesion is benign and may regress following an incisional biopsy [1].

**Table 1:** List of reports of oral melanoacanthoma [2-14]

S. no.	Author	Year	No. of patients	Site
1	Tomich [11]*	1978	1	Buccal mucosa
2	Matsuoka et al. [2]*	1979	1	Labial mucosa
3	Schneider et al. [2]	1981	1	Buccal mucosa
4	Wright et al. [2]*	1983	2	Buccal mucosa
5	Goode et al. [4]	1983	10	Buccal, labial, palatal, alveolar mucosa and gingiva
6	Frey et al. [5]	1984	1	Buccal mucosa
7	Sexton and Maize [6]	1987	3	Labial mucosa
8	Wright [2]*	1988	1	Buccal mucosa
9	Whitt et al. [3]*	1988	1	Buccal mucosa
10	Horlick et al. [3]*	1988	2	Buccal mucosa
11	Zemtsov and Bergfeld [7]	1989	1	Multiple
12	Heine et al. [2]*	1996	1	Buccal mucosa – bilateral
13	Chandler et al. [8]	1997	1	Palate
14	Flaitz [9]	2000	1	Gingiva
15	Fatazedah and Sirois [10]	2002	1	Multiple sites
16	Fornatora et al. [3]*	2003	10	Buccal (including bilateral), gingival, labial and palatal mucosa; retromolar pad, floor of the mouth
17	Buchner et al. [11]	2004	7	Buccal, labial and lingual mucosa
18	Kauzman et al. [12]	2004	1	Buccal mucosa
19	Andrews and Trask [13]	2005	1	Buccal mucosa
20	Carlos-Bregni et al. [14]	2007	4	Buccal mucosa, gingiva, palate

*Cross-referenced from the literature.

## Case presentation

**Figure 1:**
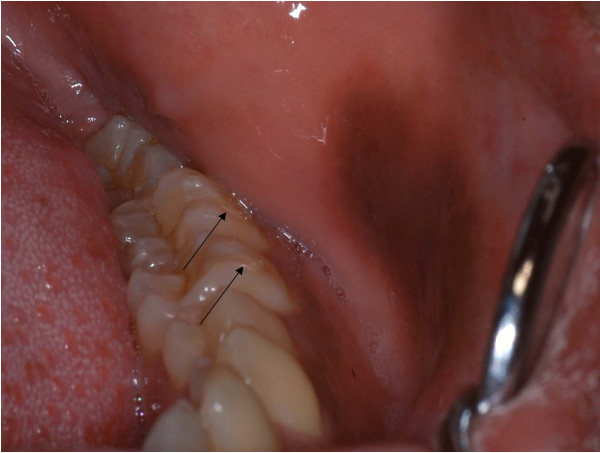
**Brownish-black macular lesion on left buccal mucosa adjacent to molar teeth with Class V glass ionomer cement restorations (arrows)**.

A 24-year-old graduate dental student presented with a complaint of intra-oral pigmentation of the left buccal mucosa with duration of one and a half months. The patient had initially noted a small round area of pigmentation of about 5 mm in size which, to his concern, had rapidly increased to the present size (Figure 1). He did not report any discomfort associated with the lesion, except for an altered surface texture. Personal history revealed that the patient had infrequently (once a day) smoked filtered cigarettes over the previous 4 years. Intra-oral examination revealed carious 28, multiple teeth with glass ionomer cement (GIC) class V restoration (36, 37, 38, 46, and 47) and a brownish-black macular lesion in the left buccal mucosa. On further enquiry, the patient revealed that he had undergone multiple GIC restorations 3 months previously, during which procedure he had sustained a mild bur injury in the left buccal mucosa, which healed uneventfully.

The brownish-black macular lesion on the left buccal mucosa was well demarcated from the surrounding mucosa with regular, well-defined borders. The lesion extended anteriorly from the region of the mandibular first molar (36) to the mandibular left canine region. It measured 25 mm antero-posteriorly and had a maximum width of 16 mm supero-inferiorly. The lesion was not tender, did not blanch under pressure and was not fixed to the underlying mucosa.

### Diagnosis

**Figure 2:**
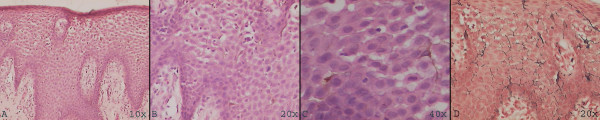
**Hematoxylin and eosin stained sections (A, B and C) revealed stratified squamous non-keratinized epithelium exhibiting acanthosis and numerous dendritic melanocytes throughout the entire thickness of the epithelium; Masson-Fontana (M-F) special stain reveals numerous melanocytes**.

Following incisional biopsy, the specimen was fixed in 10% neutral buffered formalin, routinely processed and paraffin embedded. Histopathological examination of the lesion revealed a stratified squamous surface epithelium exhibiting acanthosis, spongiosis, melanin pigmentation, inflammatory cell exocytosis and numerous dendritic melanocytes distributed diffusely in the suprabasal and spinous layers. A chronic inflammatory cell infiltrate was present in the subjacent connective tissue. The dendritic melanocytes were also demonstrated by Masson-Fontana silver impregnation stain (Figure 2). Based on the history, clinical features and histological presentation, the lesion was diagnosed as melanoacanthoma.

### Management

**Figure 3:**
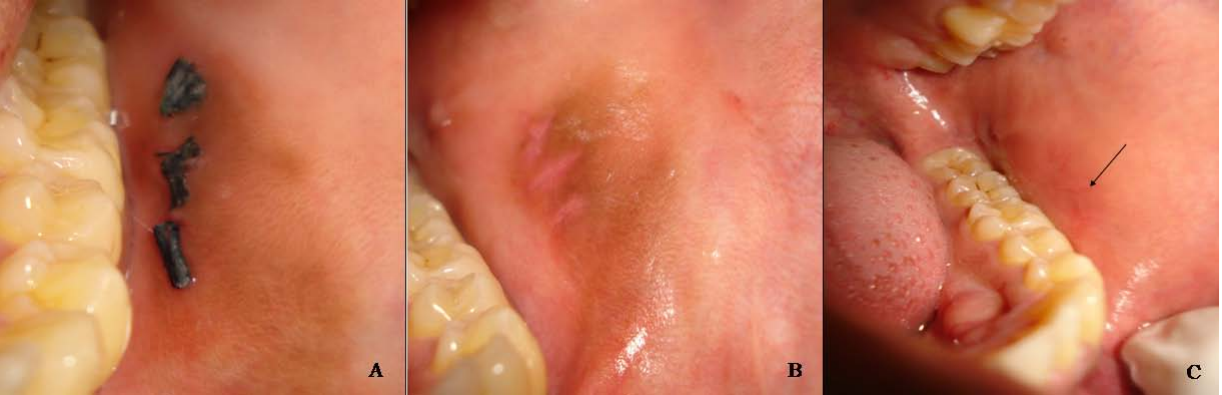
**Follow-up of lesion after 1 week (A), after 2 weeks (B) and complete resolution after 2 months (C)**.

The lesion characteristically appeared to regress following the biopsy procedure. A regular follow-up of the patient was carried out to observe the progress of the lesion (Figure 3).

## Discussion

The term melanoacanthoma refers to a lesion exhibiting a proliferation of dendritic melanocytes throughout the surface epithelium. Cutaneous melanoacanthoma is also known as pigmented seborrheic keratosis [15].

Oral melanoacanthoma is a benign, reactive process and is unrelated to cutaneous melanoacanthoma. The reported age of presentation ranges from 9 to 77 years, with a mean age of 29 years [3,4,12]. The lesion is most predominantly observed among black patients, though occurrences have been observed among Caucasians, Hispanics and Asians [1,4,12-14]. Oral melanoacanthomas show a female predilection, with a male to female ratio of 2:1 [1,2,14]. The etiology has been largely attributed to local irritation or even mild trauma [3,14]. The intra-oral site most commonly affected is the buccal mucosa but involvement of other sites such as the mucosa of the lip, palate, gingiva and alveolar mucosa has also been reported (Table 1). Clinically, the lesion is a flat or slightly raised black or brown macule and may rapidly increase in size, ranging from a few millimeters to several centimeters [1,12,13]. The lesions are usually solitary and well circumscribed though a few authors have reported bilateral or multiple (Table 1) melanoacanthomas. Oral melanoacanthomas are usually asymptomatic and are not neoplastic. The other lesions to be considered in the differential diagnosis are smoker's melanosis, drug induced pigmentation, Addison's disease, melanotic macule, pigmented nevi – junctional, intramucosal, compound, Spitz nevus, postinflammatory melanosis and oral melanoma. A biopsy is mandatory to rule out melanoma and to alleviate patient apprehension. Histologically, melanocytes which are usually restricted to the basal layer are found distributed throughout the epithelium. These melanocytes exhibit prominent dendritic processes and are immunoreactive for S-100, Melan-A/Mart-1, HMB-45 and Tyrosinase [14]. Other dendritic cells in the oral mucosa are the Langerhans' cells which are antigen presenting cells of the immune system, usually distributed in the superficial epithelium and are demonstrated on immunohistochemistry by S-100 or CD1a. The adjacent connective tissue exhibits chronic inflammatory cell infiltrate. The presence of eosinophils among the inflammatory cells is not a universal feature and may not be essential for the diagnosis of oral melanoacanthoma. Once diagnosis is established, no further treatment is required, with some cases exhibiting spontaneous regression after biopsy [1]. It has been suggested that this entity be renamed melanoacanthosis or oral melanotic macule – reactive type, since the term melanoacanthoma is suggestive of a neoplastic process [11].

In our patient, the etiology of the lesion may be attributed to the incident of trauma during the restorative procedure. It may be safely assumed that GIC did not contribute to the cause of the lesion since the patient has multiple restorations with the same material and the adjacent sites did not exhibit any lesion.

## Conclusion

To the best of our knowledge, this is the first case of oral melanoacanthoma in the Indian subcontinent and the second case of melanoacanthoma reported in an Asian Indian. In the present instance, a biopsy was performed to alleviate the patient's anxiety and as reported, the lesion regressed following biopsy. Thus, melanoacanthoma must be considered in the differential diagnosis of rapidly progressing pigmented lesions of the oral cavity and requires a histopathological diagnosis to rule out melanoma.

## Consent

Written informed consent was obtained from the patient for publication of this case report and any accompanying images. A copy of the written consent is available for review by the Editor-in-Chief of this journal.

## Competing interests

The authors declare that they have no competing interests.

## Authors' contributions

Both authors have made substantial contribution with individual input as follows:

KR was responsible for identification, diagnosis of the case, drafting of manuscript and final correction of the version to be published. VL was involved in follow up of the patient, literature review and revising and submission of manuscript. The final version of the manuscript was approved by both authors
